# Gold Nanotheranostics: Proof-of-Concept or Clinical Tool?

**DOI:** 10.3390/nano5041853

**Published:** 2015-11-03

**Authors:** Pedro Pedrosa, Raquel Vinhas, Alexandra Fernandes, Pedro V Baptista

**Affiliations:** UCIBIO, Department of Life Sciences, Faculdade de Ciências e Tecnologia, Campus Caparica, 2829-516 Caparica, Portugal; E-Mails: pm.pedrosa@campus.fct.unl.pt (P.P.); r.vinhas@fct.unl.pt (R.V.); ma.fernandes@fct.unl.pt (A.F.)

**Keywords:** nanotheranostics, diagnostics, targeted therapy, cancer, gold nanoparticles, precision medicine, theranostics

## Abstract

Nanoparticles have been making their way in biomedical applications and personalized medicine, allowing for the coupling of diagnostics and therapeutics into a single nanomaterial—nanotheranostics. Gold nanoparticles, in particular, have unique features that make them excellent nanomaterials for theranostics, enabling the integration of targeting, imaging and therapeutics in a single platform, with proven applicability in the management of heterogeneous diseases, such as cancer. In this review, we focus on gold nanoparticle-based theranostics at the lab bench, through pre-clinical and clinical stages. With few products facing clinical trials, much remains to be done to effectively assess the real benefits of nanotheranostics at the clinical level. Hence, we also discuss the efforts currently being made to translate nanotheranostics into the market, as well as their commercial impact.

## 1. Nanotheranostics: Concepts and Strategies at a Glance

Significant efforts have been made in the past years towards understanding the genetic and pathophysiological processes contributing to malignant transformation and tumorigenesis. The overwhelming amount of information thus retrieved is now being translated into the field of biomarker discovery and foster cancer therapy by selective interference with cancer hallmarks. Despite these efforts, conventional cancer therapy, including surgery, chemotherapy and radiation, lack target cell specificity and are often disconnected from individual diagnosis. Also, the efficacy of conventional therapeutic strategies is often limited by the acquisition of multidrug resistance by tumor cells and by poor drug penetration into the tumor [1]. This is why several molecular targeted therapeutics have been designed to selectively target tumor cells, the benefits being improved efficacy and decreased toxicity [2]. These novel targeted therapeutics may be engineered to simultaneously provide information about delivery, biodistribution and diagnostics, e.g., as imaging agents allowing disease detection at its early and asymptomatic stages [3]. Integration of diagnosis and therapy in a single platform has been termed theranostics, which has promised to significantly increase the precision and effectiveness of treatment, shifting the current clinical standard from generalized procedures to a personalized or precise approach [4,5,6,7]. Consequently, theranostics shows particular impact in heterogeneous diseases that require individualized and tailored methods of treatment and monitoring, such as cancer [3,8,9], rheumatoid arthritis [10], infection and cardiology [11,12]. Nanotechnology has proven its capability to engineer solutions to enhance theranostics—nanotheranostics—to simultaneously diagnose a disease and monitor therapeutic efficacy noninvasively and in real-time. This tailored approach enables physicians to customize treatment based on each patient’s responses and needs, thereby preventing unwanted deleterious side-effects or sub-optimal dosage that might lead to drug resistance, incomplete remission and relapse [3,13,14].

Nanostructures’ primary advantage is their size, ranging from 1 to 100 nm, and their augmented surface area-to-volume ratio. They can be loaded with a plethora of (bio)molecules, such as imaging moieties (e.g., fluorophores), targeting (e.g., antibodies and peptides), therapeutic agents (e.g., chemicals, siRNA, therapeutic oligonucleotides, *etc.*) and stabilizers (e.g., polyethylene glycol, PEG), which greatly favor solubility, bioavailability, and circulation half-life. These nanocarriers provide a platform for vectorization of agents, localized concentration of molecules and a protective effect upon the cargo, preventing degradation before reaching the biological target. From the available functionalization moieties, several may be easily grafted via simple chemical protocols to enhance delivery, cancer cell uptake and deep tissue penetration, ensuring time and spatial control over imaging and/or therapeutics [3]. In some cases, the therapeutic component may simultaneously provide for optical imaging, where doxorubicin is a good example with its cytotoxic effect coupled with inherent fluorescence for tissue/cell tracking [15]. Most nanosized delivery vehicles revolve around nanoparticles (NPs)—silica, gold, silver, magnetic (mainly iron oxide) and quantum dots; polymeric structures—liposomes, dendrimers, hydrogels; and carbon nanotubes [16,17].

The use of nanocarriers for theranostics has relied on the intrinsic passive targeting capability (*i.e.*, NPs tend to accumulate in cancer controlled environments derived from the altered vasculature of blood and lymph vessels that provide for a so-called enhanced permeability and retention effect, EPR), which may be selectively directed towards a particular focus via the use of active targeting moieties. Amongst the nanosized dependent properties, the optical properties of these nanocarriers have been widely explored in nanotheranostics’ application. In fact, surface plasmon resonance (SPR) resulting from photon confinement to the small particle size is a remarkable property of noble metal nanoparticles, such as gold, which has found a multitude of biological and medical applications [8,18,19,20,21,22]. Indeed, these nanosized, multifunctional platforms allow monitoring the route taken by the formulation, its delivery kinetics, intra-organ and/or intra-tumor distribution, which ultimately allow evaluation of strategy and tuning of efficacy. Nevertheless, there are still some drawbacks, such as (i) the overwhelming relevance attributed to the EPR effect; (ii) the poor tumor/tissue penetration of nanoformulations that render them ineffective due to stromal modifications; (iii) the need for a more effective targeting of metastasis; and (iv) the toxicological impact on the whole organism [1,3].

## 2. Focus on Gold

**Figure 1:**
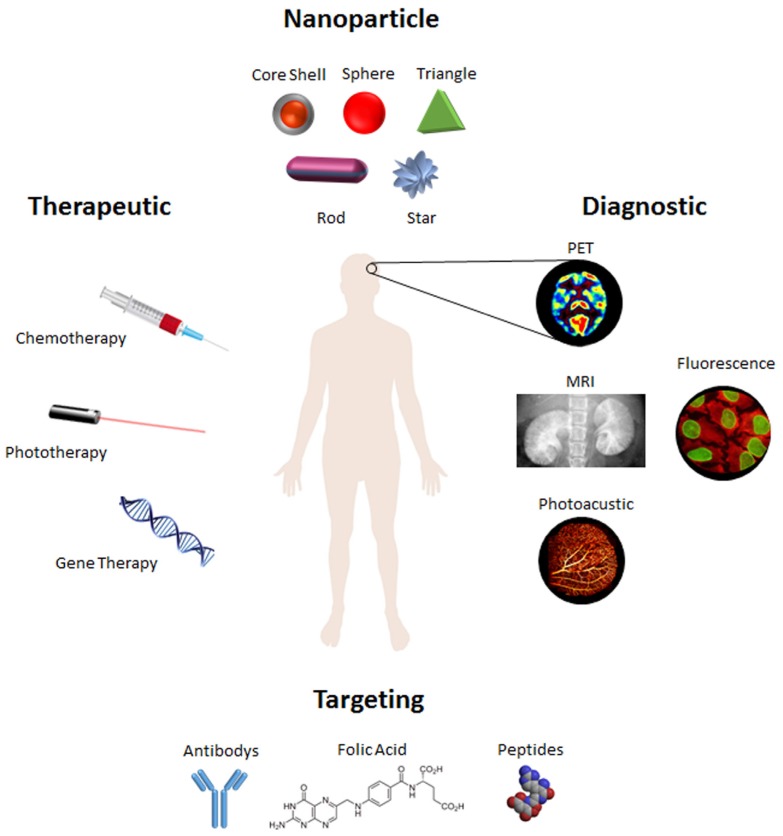
Different types of nanoparticles and their applications in theranostic. Schematic overview of the possible functionalization and application of gold nanoparticles (AuNPs) as nanocarriers for theranostics.

Gold nanoparticles (AuNPs) have been extensively studied and applied in several concepts of nanotheranostics. Their intense SPR, resulting in intense light absorption and scattering, and a high photothermal conversion rate, their ease of synthesis in a variety of sizes and shapes in aqueous media, and the fact they are easily functionalized by almost any kind of (bio)molecule recurring to simple and robust synthetic routes have turned AuNPs into powerful theranostic tools (for an overview of gold nanoparticle utilities see Figure 1) [3,23]. In addition, when compared to other types of nanoparticles, AuNPs have been considered to exhibit low toxicity and high chemical stability [17,23,24,25]. This review will focus on the use of AuNPs for *in vivo* nanotheranostics with a critical view towards the steps ahead to achieve effective clinical translation. Most deliverables of theranostic systems have focused on nanoparticles for cancer treatment, and, therefore, this review will focus on this heterogeneous disease as a model, but examples and translations to other diseases are provided.

### 2.1. Gold Nanoparticles at a Glance

Gold nanoparticles have led the world of nanotheranostics with their unique physical and chemical properties. Ranging in diameter from small clusters of 2–5 nm up to 100 nm, AuNPs can be synthesized in different shapes such as spheres, hollow, rods, diamonds, prisms, cages, either single solid bodies or in a core shell format [26,27]. Each combination of size and shape shows slightly different properties that may be explored for theranostic purposes, such as optical properties from intense bright colors, contrast agents and photothermal capability in the infrared (IR) and near infrared (NIR), biofunctionalization potential and toxicity [20,28].

Whilst the majority of the described nanotheranostics systems using AuNPs have focused on spherical NPs, shape modification will tune absorption and scattering properties towards the NIR: nanostars, nanorods, hollow nanoparticles and nanodiamonds present a high absorption peak in the NIR range, which have found plenty of applications in phototherapy approaches. For example, hollow gold nanospheres (44 nm diameter), one of the first nanotheranostics systems to be evaluated *in vivo*, have shown comparable photothermal capacity using 100 times lower concentrations to that of superparamagnetic iron oxide silica coated counterparts [29]. What is more, hollow nanoparticles and nanocages show the advantage of simultaneously acting as encapsulating vectors, allowing a higher load of agents per particle [30]. Gold nanoshells present a spherical dielectric core particle and a thin nanoscale gold shell. They are generally synthetized with silica, but different cores have been described, taking advantage of the properties of other nanomaterials, like magnetism (FeO) and high scattering (Ag) [31,32]. By controlling the thickness of the gold shell and the diameter of the core, the plasmon resonance and the resulting optical absorption of gold nanoshells can be tuned towards the NIR [33]. These NIR responsive gold nanoshells may be used as photoabsorbers for NIR photothermal ablation therapy [34]. Recently, Huang and collaborators synthetized bellflower AuNPs (GBFs), through a liquid-liquid-gas triphase interface system produced by ultrasound induced vacuum bubbles in a two-phase liquid-liquid system [35]. GBFs showed a strong plasmon band around 800 nm making them good photothermal antenna candidates for nanotheranostics applications, and their intravenous administration has been shown to delay tumor growth or promote complete tumor regression after two weeks with low laser power (0.5 W/cm^2^), while allowing photo acoustic imaging [35]. Despite the negligible toxicity described *in vitro* and *in vivo* (no weight loss in control mice), further biodistribution and toxicity studies are needed before these GFB may translate to the clinic [35].

Despite the divisive studies about AuNPs’ toxicological impact *in vivo*, spherical AuNPs between 10 and 60 nm in diameter are generally considered as non-toxic. In fact, toxicity may vary with administration route, concentration and surface coverage but also with the nanoparticles’ sizes and shapes. The difficulty in standardization of the nanoconjugates, their characterization and the effect of monitorization, has largely hampered forming a clear cut definition for *nanosafe* AuNPs [36,37]. For example, larger particles, 45 nm in size, have been demonstrated to exhibit higher cytotoxicity at a lower concentration (10 μg/mL) than those 13 nm in size (75 μg/mL) [38]. Oral and intraperitoneal administration routes showed higher toxicity than tail vein injection [39]. Sharp-edged nanoparticles tend to have higher cytotoxicity than rounded ones, with several studies showing that lower concentrations are needed for stars and flowers to induce toxicity, compared to nanospheres [40,41]. On the other hand, sharp-edged conformations are more prone to endosomal escape favoring drug delivery [42]. These factors have been extensively debated and readers are directed to those works for further and more comprehensive discussion [24,43,44,45].

### 2.2. Targeting and Delivery

AuNPs can be functionalized with molecules to provide for targeting and to enhance stability and biocompatibility *in vivo*. There is a wide range of stabilizers available that may also act as therapeutic agents (e.g., miRNA, siRNA, DNA, peptides and antibodies) or to reduce NPs’ immunogenicity (e.g., PEG). In fact, PEG of different molecular weights is one of the most frequently used biomolecules to increase NPs’ circulation half-life and improve cellular uptake [46,47]. When referring to PEG grafting to AuNPs’ surfaces, bi-functional PEGs (with a thiol group in one extremity and another group at the other end—amine, carboxylic, biotin, azide) have been widely used for direct coupling to another molecule of interest via straightforward chemistry with high yield [3,23]. AuNPs can be further engineered to trick the immune system, avoid removal from circulation (by becoming trapped in the liver, kidneys, spleen) or to cross biological barriers (e.g., blood-brain barrier), in order to increase therapeutic efficacy and allow systemic tracking [48].

Passive targeting takes advantage of the fact that vessels surrounding the tumors are leaky, due to incomplete endothelial linings, allowing nanomedicines to reach the tumor through the EPR effect alone. Generally, particles between 10 and 60 nm in diameter tend to passively accumulate in tumor tissues enabling, for instance, higher drug payload at the tumor site and circulating half-lives about 100 times longer than that of free anticancer drugs, with reduced systemic toxicity [49,50]. However, this strategy depends greatly on the degree of tumor vascularization and angiogenesis and on heterogeneous blood flow, which limit drug uptake and homogenous distribution within the tumor. Several approaches may overcome these limitations, for instance, using vasoconstrictive drugs. These agents cause normal vessels to constrict and blood pressure to increase, while tumor vessels do not respond to this effect because of insufficient muscular structure, thus leading to a relative increase in the input function of tumor tissues [51]. These leaky effects may also have an important role in the treatment of other pathologies. Spivak and co-workers developed and tested a gold nanoformulation for drug delivery and treatment of heart failure by demonstrating that levosimendan functionalized AuNPs (Simdax) were able to accumulate in the endothelial cells of infarcted arterioles and capillaries. The nanoformulation showed significant cardioprotective effects in doxorubicin-induced heart failure rats, higher than that of Simdax alone. When comparing the route of administration (intravenous injection, sonoporation—cell permeation by ultrasounds and local, and intrapleural (local delivery) injection), the best results were obtained using intrapleural injection, showing the importance of the administration route for effective treatment [52].

Another strategy relies on attaching targeting moieties to the nanoparticle surface. Typically, nanoparticles reach target cells through ligand-receptor interactions that induce receptor-mediated endocytosis and drug release inside the cell [50]. Peptide conjugation to AuNPs is one method by which active and specific targeting may be used for enhanced tumor accumulation/delivery. Chanda and collaborators used bombesin peptide-functionalized AuNPs to target the gastrin-releasing peptide (GRP) receptor which is overexpressed in breast, prostate, and small-lung carcinomas [53]. Bombesin peptide-functionalized AuNPs exhibited GRP-enhanced tumor accumulation and decreased liver uptake compared with nonspecific protein-conjugated AuNPs. In two other studies, it was possible to actively target endothelial cells of the tumor vasculature, through αvβ3 integrin, using an RGD-peptide conjugated to an AuNP. These RGD functionalized AuNPs showed an increased tumor accumulation in mice xenograft studies [54,55]. c(TNYL-RAW) and M2Pep are two examples of effective *in vivo* targeting peptides that specifically accumulate in EphB4 overexpressing cells and in tumor associated macrophages, respectively [56,57].

Successful targeting has also been achieved via functionalizing AuNPs with antibodies that target specific receptors overexpressed by cancer cells. Choi and collaborators used PEGylated AuNPs decorated with various amounts of human transferrin to provide greater intracellular accumulation within solid tumors than their non-targeted analogs [58]. Epidermal Growth Factor Receptor (EGFR) and folate receptors are two well-known overexpressed proteins in cancer cells used for designing antibody-gold nanoconjugates for active targeting [59,60]. EGFR exists on the cell surface and it is mostly activated by binding of Epidermal Growth Factor (EGF) and Transforming Growth Factor α (TGFα). Genetic mutations led to EGFR permanent activation and uncontrolled cell division independently of the EGF or TGFα presence. About 30% of epithelial cancers show misregulation in EGFR or other family members [61]. The use of Cetuximab (Erbitux^®^, Merck, Germany), an FDA approved monoclonal antibody that specifically binds EGFR and turns off its downstream signaling pathways, has been shown to be a useful targeting and therapeutic moiety in mice, vectorizing the nanoparticles to the tumor site [60,62,63,64]. Most of the accumulated particles stayed in the tumor for 72 h post tail-vein injection [59]. Others have also described the use of EGFR targeted ligands for active targeting [65], such as C225 antibody [66,67]. Using this antibody, Lukianova-Hleb and collaborators showed an increased uptake of nanoparticles by mice xenographs in EGFR-positive compared to EGFR-negative cells [67]. Van de Broek and collaborators used branched AuNPs functionalized with nanobodies targeting HER2 antigen which is highly expressed in breast and ovarian cancer cells [68].

Antibody based targeting has also been applied for theranostics of infectious diseases. Using specific antibodies against *S. aureus*, authors were able to specifically detect and kill the pathogens in the mice bloodstream [69] and in bronchoalveolae [70].

Folate receptor is responsible for the internalization of folic acid and several studies confirmed that about 80%–90% of ovarian tumors overexpress this receptor [71]. Drug-folate conjugates have therefore been at the forefront for selective tumor targeting in preclinical studies [72,73,74,75,76,77]. Lu *et al.* demonstrated that folate functionalized nanoparticles were 4.7 times more directed to tumor cells and internalized by endocytosis into lysosomes when compared to non-functionalized NPs [78].

Targeting has been crucial for the development of vectorization systems capable of site specific accumulation/retention enabling nanoparticles to deliver their cargo on site for improved therapeutic effect [49]. Some authors have been focusing their attention on developing nanovectorization systems that mediate cargo delivery upon a particular stimulus, such as pH, increased heat, ultrasound, light and magnetic field. Tumor cells usually become hypoxic and exhibit high glycolytic activity, thus producing carbonic and lactic acids [79]. Coating nanoparticles with pH Low Insertion Peptides (pHLIPs) increase efficiency of targeting acidic diseased tissues. pHLIP, being a membrane peptide, has affinity to cellular membranes and targets extracellular acidity. In contrast to other pH-sensitive systems, it tracks acidity at the surface of cancer cells [80]. Antoch and collaborators reported a 34% increase in pHLIP decorated nanoparticles in contrast with AuNPs alone, on human lung carcinoma cell lines at pH 6.0 [81]. Photothermal conversion of light into heat can increase target cells’ temperature, enough to directly kill cells [82], but also to release therapeutic moieties at the tumor site [83]. In fact, a study shows the photothermal induced release of Dox from Dox-loaded gold nanocages. By the degradation of a thermal sensitive polymer covering the NPs when the medium reaches 45 °C, DOX is released in a time dependent manner. Authors were able to significantly decrease cell viability of breast cancer cells *in vitro*, by precisely controlling DOX release [84]. In a similar manner, ultrasounds and magnetic fields can locally increase temperature and precisely deliver cargo. The reader is directed to the following references for a thorough discussion on the mechanism and detailed characterization [85,86,87]. Despite the broad range of targeting approaches, there is still much more to explore in this field with many groups still looking for new effective targeting moieties that allow tumor specific accumulation and retention for improving drug release and therapeutic efficacy [49,79].

### 2.3. Therapeutic Agents

Drug discovery coupled with preclinical studies strongly focus on overcoming general issues that hamper the efficacy of drugs, such as limited solubility, high toxicity, high dosage, nonspecific delivery and short circulating half-lives [88]. Due to the advances in nanotechnology based drug delivery, the current idea has been to repackage classic drugs using targeted delivery systems to increase patient compliance, extend the product life cycle and reduce healthcare costs. These agents might be a small drug, a peptide/antibody, a ribozyme, a siRNA or an antisense oligonucleotide [23]. Probably the most studied and used concept has been Doxil^®^ (Janssen Products, LP, Horsham, PA, USA), where doxorubicin (approved by FDA in 1974 to treat a broad range of cancer types) has shown improved efficacy when encapsulated into PEGylated liposomes. In fact, traditional chemotherapy drugs have followed suit and been encapsulated and delivered in similar engineered vesicles.

AuNPs doxorubicin nanoformulations attempted to mimic their liposome counterpart and showed promising advantages, such as increased targeting and functionalization as well as the possibility to couple with phototherapy and to act as imaging/contrast agents [89,90]. Various AuNPs shapes have been functionalized with doxorubicin—stars [54], clusters [91], shells [92], hollow spheres [57], *etc.—*showing the versatility of the nanoconstructs and their ease of functionalization. All previous examples were tested *in vivo* and have shown increased efficiency when compared with doxorubicin alone, as well as lower levels of cytotoxicity.

AuNPs have been also applied in theranostic approaches with other anti-tumor drugs (e.g., platinum (IV) prodrugs [93], 5-fluorouracil [94], irinotecan [78], camptothecin [95]). Dhar and collaborators showed a significant increase in cytotoxic effects for the Pt(IV)-AuNP complex when compared with the free Pt(IV) prodrug, as well as the free cisplatin. The Pt(IV) prodrug was activated into its cytotoxic form, cisplatin, only after crossing the cell membrane and undergoing intracellular reduction [93]. Camptothecin is a cytotoxic quinoline alkaloid which inhibits DNA topoisomerase I [96]. Camptothecin showed remarkable anticancer activity in preliminary clinical trials for a broad spectrum of tumors but also low solubility and adverse effects. Shi and collaborators described the use of gold hollow and nanocage shells layered with mesoporous silica and a thermosensitive polymer loaded with Camptothecin. The drug is tightly packed inside the nanoconstruct with a “leakage” of only 6.8% to the medium after 14 h in *in vitro* conditions. NIR triggered release of the drug leading to increased tumor cell death, compared with the only NIR and nanocarrier experiment [95]. For rheumatoid arthritis treatment, iron-gold nanoparticles have been functionalized with methotrexate (MTX) allowing for simultaneous chemo-phototherapy and imaging with lower dose (0.05%) without compromising efficacy and/or increasing toxicity [97].

Gene therapy has been receiving increasing attention in tumor suppression due to the possibility to downregulate specific oncogene expression or to sensitize cells in an intra-cellular targeting process. In particular, small interfering RNA (siRNA) has shown potential to downregulate specific gene expression in cancer cells [25]. Since naked siRNAs show extremely short half-lives due to cellular RNases activity and poor chemical stability, the development of efficient delivery vehicles for *in vivo* applications remains a major obstacle in translating siRNA into effective therapeutics. AuNPs have been widely used as nanovectorization for gene silencing strategies [98,99,100,101,102,103]. Almost all the different shapes and sizes of AuNPs reported above have been used to vectorize gene silencing elements into cancer cells [104,105,106].

Wei Lu *et al.* reported in 2009 the use of hollow gold nanospheres carrying siRNA recognizing NF-κB p65 subunit. NF-κB has been linked to tumor formation and progression, increasing the expression of antiapoptotic and survival factors and inhibiting apoptosis. Increased sensitivity to chemotherapeutic agents such as irinotecan has been associated with inhibition of NF-κB. After intravenous injection of the nanoconstruct with the siRNA, significant downregulation of the NF-κB p65 subunit was observed in HeLa xenografts irradiated with NIR laser (800 nm) but not in non-exposed contralateral tumors in the same mice. The authors proved that the escape of the nanoparticles from the lysosomes’ and siRNA’s release in the cytosol was irradiation dependent, coining this method as “photothermal transfection” [29]. Due to their NIR band, gold rods have also been used for photothermal transfection [107] coupled to nanoparticle imaging [108], hyperthermia [109], or co-delivery [101,110]. Feng Yin reported the use of a light-triggered therapy using gold nanorods as nanocarriers for dual-delivery of doxorubicin and *KRAS* gene siRNA. The synergistic effect of the chemo- and gene-therapy allowed the reduction of the tumor volume rate by 90% *in vivo* [110]. Gold spheres were described for siRNA targeting and delivery [111,112] using models of increasing complexity (cells, hydra and mice), achieving effective silencing of *c-MYC* gene (~65% reduction in expression). It was also shown that covalently bounded siRNAs were more effective silencers than siRNAs adsorbed on the surface of the NPs [112]. Different molecular concepts for gene silencing rather than siRNA have also been used to block a particular gene.

**Figure 2:**
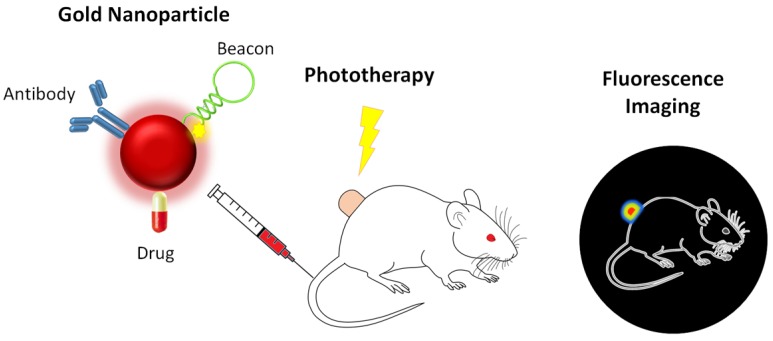
Schematics of a multifunctional approach, coupling targeting, chemotherapy, gene therapy, phototherapy and diagnostics by fluorescent imaging.

Baptista’s Group was pioneer in the use of hairpin ssDNA structures vectorized via AuNPs to particularly silence any possible RNA mediated pathway inside the cell [103,113,114]. In fact, the molecular actuator thus developed—gold nanobeacon (Au-nanobeacon)—is one of the most effective molecular nanotheranostics platforms (See Figure 2), since the hairpin may be further functionalized with a fluorophore, whose fluorescence is triggered once the silencing event occurs. The potential of this system has been further characterized *in vitro* and *in vivo* with low toxicity [112,115,116]. Furthermore, this system was shown to be effective *in vivo* in a promising approach to combat multi-drug resistant tumors, combining in the same particle an antitumor agent (5-fluorouracil) with silencing of *MRP1*, a gene associated with acquired resistance in several tumors. *In vivo* results showed a remarkable tumor size reduction, from the synergic effect of the two agents [94]. Using gold nanobeacons, Bao *et al.* were able to silence *KRAS* gene and reduce gastric tumour size in mice by 60%, tumor vascularization by 90% and lung metastasis by 80% [98].

### 2.4. Phototherapy

Photothermal therapy (PTT) is based on the selective sensitization of cells to thermal damage, or hyperthermia, near 45 °C. What is more, traditional PTT may be coupled to AuNPs and profit two ways: (i) the possibility to vectorize additional cargo and use the NP as scaffold for selective targeting; and (ii) enhance PTT by the AuNPs’ ability to convert absorbed photons into thermal energy. By modulating the AuNPs’ shape and/or shell thickness, it is possible to shift the LSPR peak of AuNPs toward the near-infrared (NIR), allowing deeper light penetration into tissues. Moreover, the laser energy required to achieve this transformation is far below that stipulated in medical safety standards. AuNP-mediated PTT is predominantly associated with nanorods, nanoshells and nanocages [20,117]. Recently, Liu *et al.* developed a strategy with huge potential to be translated into cancer theranostics [117]. The successful strategy incorporates several components on a PEGylated nanorod structure: mesoporous silica coating, that enhances its drug load capacity; a tumor targeting peptide, tLyp1; and indocyanine green, an FDA-approved NIR fluorochrome. Hence, the obtained nanomaterial enabled the targeting of the fluorescent probe and of the PTT agent to tumor cells, allowing for simultaneous *in vitro* imaging and apoptosis of breast cancer cells. Before conducting the PTT experiments, the nanoconjugates showed negligible cytotoxicity. Feng *et al.* also developed a system for the targeted photothermal and chemotherapy of highly aggressive triple negative breast cancer [118]. For that purpose, they functionalized gold nanorods with chemotoxic cisplatin, biocompatible polypeptide poly-l-glutamic acid (PGA) and tumor targeting folic acid. When administered systemically on tumor-bearing mice, and in combination with localized NIR laser, the resulting hybrid nanoparticles were able to significantly inhibit tumor growth and dissemination of cancer cells from the primary site to the lung by eliminating the peripheral tumor blood vessels (a similar approach is depicted in Figure 2). Taking advantage of the magnetic properties of SPIOs and the strong NIR absorption property of gold nanoshells, Bai and colleagues developed a structure capable of simultaneously targeting (using FA) both imaging and PTT agents to cancer cells, for an effective diagnosis and therapy [119].

AuNPs have also been used to enhance plasmonic nanobubbles (PNBs) for single cell therapy [120]. PNBs are generated due to the interaction of short, high-energy optical pulses and plasmonic NPs. The interaction results in the evaporation of a thin layer of the surrounding medium, creating a vapor nanobubble that expands and then collapses in nanoseconds. The collapse of the nanobubble generates a shock wave that propagates through the medium and is proportional to the intensity of the laser pulse [66]. Depending on the laser intensity, the precision of the shockwaves can disrupt lysosomes—promoting lysosomal escape—or cellular membranes leading to cell death. PNB have therefore a mechanical effect by nature and not a thermal one. The PNB have also interesting optical properties, greatly enhancing light scattering around the nanoparticle, and have increased photoacoustic signal, making them good imaging probes for diagnostics. With proven results *in vivo*, with single cell precision, the PNB approach is a valuable tool in theranostics allowing diagnostics and therapy [66,67,120,121].

Another interesting strategy that can be coupled to nanosized structures is photodynamic therapy (PTD), which involves the administration of a nontoxic agent that acts as a photosensitizer (PS) and a laser source. Photoexcitation of the photosensitizer leads to the generation of free radicals, which destroy tumor tissue. The chosen wavelength of irradiating light should ideally be one that is absorbed to a greater degree by the tumor tissue relative to the surrounding healthy tissue [65,122]. Combining PDT with AuNPs has proven to be a good strategy to overcome two of its major limitations: the dark toxicity of the PS and the poor selectivity of the cellular uptake of PS between the target cells and normal tissues. In fact, a study describes the targeted delivery of AuNPs functionalized with EGF peptide and the photodynamic agent Pc 4. *In vitro* experiments show that the nanoconjugate is two-fold more efficient at killing tumor cells than free Pc 4, due to enhanced localization in early endosomes [65]. In another research work, AuNPs were functionalized with: methylene blue (MB), a known PS; polystyrene-alt-maleic acid (PSMA), a polymer that prevents methylene blue leakage; and transferrin (Tf), for specific receptor-mediated endocytosis. Au@polymer/MB-Tf were excited by a dark red light source at 660 nm and showed a two-fold enhancement of PDT efficiency toward *in vitro* cervical cancer cells over the use of free MB at four times dosage. Besides being an effective and easy way to induce cell apoptosis, this system proved to be safe since no significant dark toxicity was found [123].

### 2.5. Multimodal Imaging

The implementation of imaging techniques for diagnostic purposes enables the non-invasive assessment of anatomical, functional and molecular information, with image-guided drug delivery gaining much attention nowadays [124]. The imaging modalities most often used in the clinics are computed tomography (CT), ultrasound (US), magnetic resonance imaging (MRI), positron emission tomography (PET), single-photon emission computed tomography (SPECT), photoacoustic tomography (PAT) and fluorescence imaging [125]. When choosing an imaging technique, one should take into consideration several parameters: target tissue, resolution, sensitivity, contrast and implementation. CT is a commonly used diagnostic imaging tool offering broad availability at a relatively modest cost. This modality usually provides image contrast to visualize tissue density differences and may be tuned to distinguish between normal and cancerous tissue. However, the iodinated molecules that are typically used as CT contrast enhancers tend to undergo a rapid renal clearance and nonspecific vascular permeation causing a decrease in the technique sensitivity. MRI displays high spatial resolution and soft tissue contrast, but is expensive and time-consuming. US imaging offers high resolution at much lower cost than MRI or PET/SPECT, but has low depth penetration. PET/SPECT are particularly suited for targeted *in vivo* molecular imaging and their advantages include high sensitivity, absence of tissue penetration limit, and the ability to make quantitative measurements, however, these techniques expose the patient to ionizing radiation. PAT allows the reconstruction of images with improved spatial resolution and excellent image contrast when compared to conventional optical imaging [125,126,127,128,129].

Imaging techniques can take advantage of the photoacoustic phenomenon that is generated upon formation of the previously described nanobubbles. Shao *et al.* describe the use of this technique for gold nanoparticle tracking in mice, observing individual gold clusters, however, it works best on soft tissues and has depth-of-field limitations [130]. AuNPs are also optimal contrast agents for CT, due to the relatively high X-ray attenuation of gold and the stability of gold colloids. Since gold has higher absorption than iodine, AuNPs can achieve better contrast with lower X-ray dose [9]. On the contrary, AuNPs do not possess intrinsic properties that enable their visualization through MRI; hence, they are usually combined with super-paramagnetic iron oxide (SPIO), gadolinium or manganese, demonstrating significant contrast enhancement in tumor models [97]. Furthermore, *in vivo* studies with gold nanorods labelled with radioactive iodine enabled photothermal therapy towards ovarian cancer and monitoring of nanoparticles’ distribution via SPECT/CT imaging. Tumor sites were clearly visualized from the SPECT images even at 24 h post-injection [131].

Fluorescence imaging is highly suitable for high-throughput screening with high sensitivity, providing detailed molecular profiling with subcellular resolution, enabling multicolor imaging and being relatively inexpensive, but has low tissue penetration and spatial resolution, thus limiting its applications in clinical settings. Using the NIR part of the spectrum for fluorescence-based imaging, spatial resolution is improved and autofluorescence is highly reduced making the technique much more attractive for clinical applications [132]. Indeed, a study published by Topete and colleagues combined chemo-, photo-, and thermotherapies with fluorescence imaging capability for diagnosis under NIR light illumination. For that purpose, they developed a nanoplatform consisting of: a core biodegradable matrix loaded with Dox; a gold nanoshell, required for thermal therapy and drug encapsulation/release; and an outer layer consisting of plasma protein human serum albumin, for *stealthiness*, to which the fluorescent dye indocyanine green was conjugated, thus providing the nanostructure the capability to emit fluorescence in the NIR range, but also to produce singlet oxygen species (essential for the photodynamic therapy); finally, folic acid was also covalently linked to the protein surface for targeting. The effectiveness of the contrast agent was clearly enhanced by positioning the dye near the metal surface while tuning the plasmon resonance of the nanoshell to the emission wavelength of the fluorophore. *In vivo* fluorescence imaging using tumor-bearing mice demonstrated that the nanoconjugates are localized and retained in the tumor region for at least 48 h. Moreover, a larger fluorescence emission was perceived when using these nanostructures in comparison with the images obtained with similar NPs synthesized without the gold nanoshell component [75].

Owing to their underlying physical principles and distinct benefits/drawbacks, one can take advantage of combining two or more techniques in a single nanocarrier, avoiding a repeated challenge to the patient’s immune system and providing more accurate and dependable data on the patient’s condition than using a single imaging moiety [133,134]. Liu *et al.* report the use of gold nanostars for multimodal imaging. Combining CT, surface-enhanced Raman scattering (SERS, of molecules adsorbing on a noble metal surface) and thermal imaging, authors could accurately detect gold stars located in the tumor and further confirm photothermal effectiveness with a thermal camera [135]. A distinct work describes the *in situ* monitoring of doxorubicin release from gold nanoparticles, modified with a breast cancer targeting antibody (HER2) and a cell-penetrating peptide (Tat peptide) in SK-BR-3 cells, using SERS. The intracellular release of Dox from the nanoparticle was continuously monitored with time-dependent change in SERS signals, and was associated with a decrease of cancer cells’ viability, thus proving to be a promising theranostic approach to manage breast cancer [136]. Other successful examples of combined imaging modalities using gold nanoparticles include: a multicomponent nanocapsule used for real-time US and high resolution MRI for image guided photothermal tumor ablation [34]. Also, gold quantum dots within a mesoporous silica shell significantly reduced tumor growth *in vivo* while coupling three complementary imaging modalities: NIR fluorescence, photoacoustic, and magnetic resonance imaging. [137]. Arifi *et al.* proposed a trimodal Gadolinium-Gold capsule for ultrasound, CT and Positive-Contrast MRI directed towards visualization of transplanted islet cells in diabetes treatment. These capsules showed to be not only versatile platforms for imaging but also for treatment, since Gadolinium-Gold injected mice returned healthy blood glucose levels seven days after injection and remained normoglycemic for at least six weeks [138].

Since the challenges of efficient tumor treatment include accurately identifying the location and size of tumors and monitoring the effectiveness of therapy after treatment, integration of contrast-enhanced diagnostic imaging capability with photothermal therapy is a winning bet in the fight against cancer.

## 3. From Research Lab to the Clinic

The use of gold in medicine dates back to 2500 BC when Chinese and Arabic physicians used gold preparations in their practice. Medieval physicians used mixtures of colloidal gold for various ulcerative skin conditions. In the 20th century, several formulations of gold salts were used to treat tuberculosis, *Lupus vulgarus*, syphilis and rheumatism. In 1997, Guy Abraham and Peter Himmel reported the use of 20 nm gold nanoparticles for rheumatism treatment in 10 patients with doses of 30 mg [139]. Their results showed a “rapid and dramatic” positive effect on the tenderness and swelling of joints with no evidence of toxicity in any of the patients. Since then, there have been no follow up studies on AuNPs for rheumatism in clinical settings.

Recombinant human tumor necrosis factor alpha (rhTNF) was applied in the 80s with remarkable antitumor effects in mice, inducing apoptosis, cytolysis or cytostasis of tumor cells. However, multiple phase II studies reports with more than 156 patients, resulted in only one complete and one partial response at the maximum tolerated dose, which may correlate to low therapeutic dose at disease site. CYT-6091 from Cytimmune was the first product on clinical trial using AuNPs for patients with advanced solid tumors (NCT00356980, NCT00436410). The nanoformulation is composed of PEGylated 27 nm gold nanoparticles functionalized with rhTNF. It was administered systemically to 30 patients at doses of rhTNF that were previously shown to be toxic, without detectable side effects [140]. In addition, gold was found in breast tumor tissue but not in healthy breast tissue showing the potential of this approach. Phase II clinical trials are ongoing, aiming at understanding if this nanotechnology approach induces greater vascular leak by dynamic contrast-enhanced MRI and to evaluate the safety and efficacy of CYT-6091.

AuroLase^®^ is an FDA-approved pilot study that uses silica-gold nanoshells with a NIR laser for photothermal therapy (NCT00848042, NCT01679470). Nanoparticles called AuroShells are injected intravenously in the patient’s blood stream and accumulate passively in the tumor. This clinical trial was designed to evaluate the approach’s effectiveness for the treatment of advanced lung tumors resulting from either primary lung cancer or metastatic tumors in the lung. Although the trials are complete, the results have not yet been disseminated.

The results for NANOM FIM, a completed phase III clinical trial (NCT01270139) using silica-gold nanoparticles, were recently revealed. Developed for the treatment of coronary atherosclerosis, two approaches were taken: core-shell silica-AuNP group and core-shell silica-AuNP iron bearing group. For the former, NPs were administered via surgical inclusion of a patch composed of a sliced porous bovine pericardium biological scaffold with inserted growing multilayered purified allogeneic stem cells with the initial mesenchymal phenotype. NPs were activated with an NIR laser at seven days after the intervention using a plasmon photothermal strategy. For the latter, the iron bearing NPs were managed with intracoronary infusion of allogenious stem cells and CD68 targeted micro-bubbles. CD68 targeted micro-bubbles were destroyed by ultrasounds and the nanoparticles were magnetically targeted to the vessel and lesion. NPs were also detonated with the NIR laser at the end of the procedure under the protection of anti-platelet therapy. For therapeutic analysis, quantitative coronary angiography (QCA) and intravascular ultrasound (IVUS) were performed pre-, post-procedure and at a 12 month follow-up. The results showed 12.6% reduction in the percentage atheroma volume (PAV) post procedure, 44.8% in the 12 month follow up for the first group, and 13.1% PAV reduction post procedure, and 44.8% in the 12 month follow up for the second group. The plasmonic resonance therapy using silica-gold NP proved efficient regression of coronary atherosclerosis in both approaches with acceptable levels of safety [141].

### *Commercial Impact* 

Nanotechnology R&D continues to grow in importance both in terms of public and private funding. Indeed, global demand for nanotechnology medical products grew by 17% from 2009 to 2014 [142]. The United States (U.S.) National Nanotechnology Initiative (NNI) investment in nanotechnology R&D, policy and regulation since 2001 now totals a staggering $22 billion, with the 2016 U.S. federal budget providing more than $1.5 billion for the NNI [143]. The European Union and Japan are investing substantial resources in nanotechnology, with a comparable level to the U.S. [144]. The returns are expected to surpass the investment, and the medical field appears to be a front runner in nanotechnology innovations. Nanoparticle based drug delivery is one of the major areas, providing a wide range of formulations that are now beginning pre-clinical or clinical trials. Besides the traditional hurdles conventional therapeutics face to enter the clinics, nanomedicines also face a lack (or the deficiency) of protocols for the characterization of these products in terms of absorption, distribution, metabolism, excretion, and toxicity [145,146]. However, the FDA, in collaboration with the NCL (Nanotechnology Characterization Laboratory), has released guidelines describing how it defines a nanoscale product—specifically, as having at least one dimension between 1 and 100 nm, or as being less than a micron in size, and demonstrating size-dependent behavior. These agencies are now drafting protocols to address nanomedicines’ regulatory and safety gap. The European Commission has also established several goals in terms of nanotechnology regulation, patenting and business creation, with international cooperation being a key asset to improving R&D. Bringing the EU together with countries who are active in nanotechnology research (USA, Japan, Switzerland and Russia) could pave the way for standardized protocols and further initiatives [144]. The fact that nanotechnology is one of the sections included in the Horizon 2020, the biggest EU Research and Innovation Programme, shows the commitment to this subject and its importance in increasing Europe’s competitiveness. Its main objectives include: scaling up laboratory experience to industrial settings; ensuring the safe development and application of nanotechnologies in health and the environment; and proving the significant long term benefits provided by nanotechnology-based systems, in terms of health care and quality of life [147].

Nanomedicines are still at an early development stage and, thus, their impact on health spending and cost effectiveness is still difficult to predict. Can these formulations compete with conventional products in the medical sector? For example, examining the cost-effectiveness of chemotherapy (gemcitabine) *vs.* nanotherapy (PEGylated liposomal doxorubicin) for ovarian cancer showed that the chemotherapy pre-treatment costs were cheaper by €1285. Nonetheless, these costs were more than offset by administration and hospitalization costs, which were €2670 in favor of nanotherapy. The clinical benefit associated with nanotherapy was proven, yielding not only positive cost-effectiveness results, but also significant financial savings [148].

Nanotheranostics innovations, in particular, can have a significant impact on health costs by reducing the amount of diagnostic tests and increasing therapy efficacy. Additional savings are expected due to the overall reduction of the number of days in hospital for each patient. Hence, the global market for nanomaterials used in theranostics is anticipated to be more than $187 billion in 2017 [149]. Despite these figures and expectations, for an overview of these accounts for all the nanoformulations reaching the (pre-) clinical stage, where those relating to AuNPs alone are just the tip of the iceberg—see Table 1. Another good indicator of AuNPs’ increasing impact on the medical sector is the number of companies that are devoted to R&D in this area alone. Due to high competition among the market players and low yield coupled with high expenditures in R&D, most companies choose to invest in specific end-use applications: diagnostic tests for the point-of-care market, biomedical imaging or photothermal therapy. Successful examples include Nanospectra (Aurolase^®^) and Cytimmune (Aurimune^®^) whose flagship products are now facing clinical trials. Although the market for gold nanomaterials is still developing, their clinical and financial benefits are undeniable. In the future, efforts should be made to improve the bridge between academia, R&D companies, regulatory agencies and the pharmaceutical industry. In Table 1, we compiled a brief list of the theranostic strategies using AuNPs currently in pre-clinical and clinical studies.

**Table 1:** Compilation of gold strategies. Brief overview of current efforts in AuNPs for theranostics–pre-clinical and clinical studies.

Particle	Targeting	Therapeutic	Phototherapy	Imaging	Refs
**PRE-CLINICAL STUDIES**					
Silica-Gold Shells (150 nm)	-	-	Photothermal	MR	[150]
Gold Hollow Spheres (40 nm)	Melanocortin Type-1 Receptor	-	Photothermal	PET	[29]
Gold Spheres (60 nm)	EGFR	-	PNB	Scattering	[66]
Gold Hollow Spheres (40–50 nm)	Folate Receptor	Irinotecan + siRNA NF-κB p65 subunit	-	PET	[78]
Gold Cages (48 nm)	-		Photothermal	PET	[83]
Gold Clusters (1 nm)	Folate Receptor	Doxorubicin	-	Fluorescence	[91]
Gold Hollow Spheres (37 nm)	Ephrin Type-B Receptor 4	Doxorubicin	Photothermal	SPECT	[57]
Gold Stars (25 nm)	RGD	Doxorubicin	Photothermal	Fluorescence	[54]
Iron Oxide-Gold Spheres (6–18 nm)	A33 Antigen	-	Photothermal	MR	[151]
Gold Spheres (33 nm)	-	Tumor Necrosis Factor α	Photothermal	Photoacoustic	[130]
Gold Spheres (90 nm)	EGFR	Cetuximab	-	Raman Scattering	[64]
Gold Spheres (60 nm)	EGFR	Doxorubicin	PNB	Photoacoustic	[67]
Gold Spheres (5 nm)	EGFR	PC 4	Photodynamic	Fluorescence	[65]
Gold-Cage (40–50 nm) + Silica Sphere Shell (50 nm)	-	Camptothecin	Photothermal	Fluorescence	[95]
PLGA-Gold Shell	Folate Receptor	Doxorubicin	Photodynamic;	Fluorescence	[96]
Gold Spheres (14 nm)	-	-	Photothermal	X-ray Computed Tomography	[152]
PLGA-Iron Oxide-Gold Shells (374 nm)	-	-	Photothermal	US; MR	[34]
Gold Spheres (3.3 nm)	Folate Receptor	α-Tocopheryl Succinate	-	X-ray Computed Tomography	[153]
Iron Oxide + Gold Clusters (150 nm)	Magnetic	Doxorubicin	Photothermal	MR	[154]
PLGA-Gold Shell (115 nm)	-	Doxorubicin	Photothermal	MR	[92]
Gold Rods + Liposome Hybrid	-	siRNA PLK1	-	Multispectral Optoacoustic Tomography	[108]
Gold Bellflowers (180 nm)	-	-	Photothermal	Photoacoustic; US	[35]
Gold-Silica Rattles (150 nm)	-	Doxorubicin	Photothermal	Fluorescence; MR; Photoacoustic	[137]
Gold (20 nm) Gelatin shell (150 nm)	RGD	Doxorubicin	-	Fluorescence	[55]
Gold Stars (70 nm)	-	-	Photothermal	Thermal	[155]
Gold Spheres (12 nm); Gold Stars (30 nm; 60 nm)	-	-	Photothermal	SERS; X-ray CT; Two Photon Luminescence	[135]
Gold Rods (10:37 nm)	Folate Receptor	-	Photoacoustic	Photoacoustic	[77]
Gold Spheres (15 nm)	Scavenger Receptor (TAM)	siRNA Vascular endothelial growth factor	-	Fluorescence	[56]
Gold Rods (22:47 nm)	-	Doxorubicin + siRNA K-Ras	Photothermal	Fluorescence	[110]
Gold Spheres (15 nm)	-	Antisense K-Ras	-	Fluorescence	[98]
Gold Spheres (14 nm)	-	U5′-fluorouracile + siRNA MRP1	-	Fluorescence	[94]
PLGA-Gold-Iron-Gold	RGD; Magnetic	Methotrexate	Photothermal	NIR; MR	[10]
Gold Spheres (30 nm)	Sonoporation	Levosimendan	-	US	[52]
Gold Rods	*S. aureus*: protein A; lipoprotein	-	Photothermal	Photoacoustic	[69]
Gold-Silver Core Shell (20 nm)	Anti-*MRSA* antibody	-	-	X-ray Computed Tomography	[70]
Gold Rods (10:33 nm)	Folate Receptor	-	Photothermal	SPECT; X-ray CT	[131]
Gadolinium-Gold (2–2.5 nm)	-	Healthy Pancreatic Islet Cells	-	MR; US; Computed Tomography	[138]
**CLINICAL STUDIES**					
Gold Spheres (20 nm)	-	-	-	-	[139]
Gold Spheres (27 nm)	-	Tumor Necrosis Factor α	-	-	[140]
Silica-Gold Shell (60–15 nm; 70–40 nm)	-	-	Photothermal	US	[141]

Notes: MR, Magnetic Resonance; PET, Positron Emission Tomography; SPECT, Single-Photon Emission Computed Tomography; US, Ultra Sound; NIR, Near Infrared; EGFR, Epidermal Growth Factor Receptor; PNB, Plasmonic Nanobubble.

## 4. Final Remarks

Although much remains to be done to translate nanoformulations into clinical practice, the potential of theranostic nanoparticles is tremendous. Still in its early stages, nanotheranostics is steadily gaining the attention of traditional biopharma and clinical players. Several issues, from nanotoxicity to efficacy and precision therapy to efficient biomarkers, are still unclear and require focused research and attention. Nevertheless, it is clear that nanotheranostics probes possess several advantages when compared to conventional approaches, where diagnostic and treatment procedures are frequently disconnected and do not take into consideration the variables at stake for each patient. One issue to keep in mind is the relationship between *in vitro* and *in vivo* models and how data translates into the clinic. The urgency to push forward these nanotheranostics has sometimes resulted in the bypass of critical steps in the thorough characterization of nanomaterials, their intrinsic properties and the relationship between the lab and real patients. The novelty (often the concern of researchers) is overshadowing the solid acquisition of knowledge for a given nanotheranostics platform, and comprehensive assessment of all the AuNP based approaches might lead to the discovery of a few extremely well characterized and effective solutions that have been put aside due to the tremendous costs of pushing them through the “death valley” of advanced clinical trials. Being cancer the main target when designing nanotheranostics approaches, one of the flaws in research studies is the disconnection between mice models and human models. The need to use an immunocompromised model in a disease highly modulated by the immune system leads to the failure of most clinical trial attempts. Most clinical trials are conducted with patients with advance staged cancer where the nanoformulations seem to have high failure rates despite presenting good results for early stages of the disease. For these reasons, the whole process of pre-clinical studies, clinical trial design, and product translation should be rethought in order to realize the potential of nanotheranostics in our daily life.
